# Retraction: Metagenomic analysis exploring taxonomic and functional diversity of bacterial communities of a Himalayan urban fresh water lake

**DOI:** 10.1371/journal.pone.0275944

**Published:** 2022-10-21

**Authors:** The PLOS ONE Editors

The *PLOS ONE* Editors retract this article [1] because it was identified as one of a series of submissions for which we have concerns about authorship, competing interests, and peer review. We regret that the issues were not addressed prior to the article’s publication.

TA, GG, AS, BK, and MAES did not agree with retraction. MNA responded but expressed neither agreement nor disagreement with the editorial decision.

## References

[1] Ahmad T, Gupta G, Sharma A, Kaur B, El-Sheikh MA, Alyemeni MN (2021) Metagenomic analysis exploring taxonomic and functional diversity of bacterial communities of a Himalayan urban fresh water lake. PLoS ONE 16(3): e0248116. 10.1371/journal.pone.0248116 33764980 PMC7993826

